# Does the lipid-lowering peroxisome proliferator-activated receptors ligand bezafibrate prevent colon cancer in patients with coronary artery disease?

**DOI:** 10.1186/1475-2840-7-18

**Published:** 2008-06-19

**Authors:** Alexander Tenenbaum, Valentina Boyko, Enrique Z Fisman, Ilan Goldenberg, Yehuda Adler, Micha S Feinberg, Michael Motro, David Tanne, Joseph Shemesh, Ehud Schwammenthal, Solomon Behar

**Affiliations:** 1 Cardiac Rehabilitation Institute, the Chaim Sheba Medical Center, Tel-Hashomer, affiliated with the Sackler Faculty of Medicine, Tel-Aviv University, Tel-Aviv, Israel; 2 Bezafibrate Infarction Prevention Study Coordinating Center, Neufeld Cardiac Research Institute, the Chaim Sheba Medical Center, Tel-Hashomer, affiliated with the Sackler Faculty of Medicine, Tel-Aviv University, Tel-Aviv, Israel

## Abstract

**Background:**

Epidemiologic studies have suggested that hypertriglyceridemia and insulin resistance are related to the development of colon cancer. Nuclear peroxisome proliferator-activated receptors (PPAR), which play a central role in lipid and glucose metabolism, had been hypothesized as being involved in colon cancerogenesis. In animal studies the lipid-lowering PPAR ligand bezafibrate suppressed colonic tumors. However, the effect of bezafibrate on colon cancer development in humans is unknown. Therefore, we proposed to investigate a possible preventive effect of bezafibrate on the development of colon cancer in patients with coronary artery disease during a 6-year follow-up.

**Methods:**

Our population included 3011 patients without any cancer diagnosis who were enrolled in the randomized, double blind Bezafibrate Infarction Prevention (BIP) Study. The patients received either 400 mg of bezafibrate retard (1506 patients) or placebo (1505 patients) once a day. Cancer incidence data were obtained by matching a subject's identification numbers with the National Cancer Registry. Each matched record was checked for correct identification.

**Results:**

Development of new cancer (all types) was recorded in 177 patients: in 79 (5.25%) patients from the bezafibrate group vs. 98 (6.51%) from the placebo group. Development of colon cancer was recorded in 25 patients: in 8 (0.53%) patients from the bezafibrate group vs. 17 (1.13%) from the placebo group, (Fisher's exact test: one side p = 0.05; two side p = 0.07).

A difference in the incidence of cancer was only detectable after a 4 year lag and progressively increased with continued follow-up. On multivariable analysis the colon cancer risk in patients who received bezafibrate tended to be lower with a hazard ratio of 0.47 and 95% confidence interval 0.2–1.1.

**Conclusion:**

Our data, derived from patients with coronary artery disease, support the hypothesis regarding a possible preventive effect of bezafibrate on the development of colon cancer.

## Background

Colon cancer is one of the leading forms of malignancy in the developed countries. Epidemiologic and animal studies have suggested that risk factors for coronary artery disease like insulin resistance and dyslipidemia are probably related to the development of colon cancer [1-7]. Particularly, nuclear peroxisome proliferator-activated receptors (PPAR), mainly alpha and gamma, which play a central role in lipid and glucose metabolism, had been hypothesized as being involved in colon cancerogenesis [8-12]. Furthermore, synthetic PPAR ligands (glitazones and bezafibrate) with proven beneficial effects on insulin resistance and triglyceride levels had been proposed to be candidates as tumor preventive agents [12-14].

While dietary administration of pan-PPAR ligand bezafibrate has been demonstrated to suppress the development of colonic tumors in rodents [12-14], its effect on colon cancer development in humans is unknown. We therefore sought to investigate a potential preventive effect of bezafibrate on the development of colon cancer in patients with coronary artery disease enrolled in the randomized, double blind Bezafibrate Infarction Prevention (BIP) Study.

## Methods

The major inclusion and exclusion criteria for the BIP study, as well as the ethical guidelines, have been previously reported [15]. In brief, inclusion criteria for men and women comprised: age 45–74 years, history of myocardial infarction no less than 6 months and not more than 5 years prior to enrollment into the study and/or stable angina pectoris. The major exclusion criteria for the BIP study were: malignant diseases, permanent pacemaker implantation, cerebrovascular disease, chronic hepatic or renal disease, peripheral vascular disease, estrogen replacement therapy, insulin dependent diabetes mellitus and current use of a lipid modifying drug.

There were 3090 patients who were included in the BIP study after screening. Patients in whom a diagnosis of cancer had been made after screening but before the launch of the study medication as well as patients with unknown vital status were excluded from this analysis. Thus, the final study sample for our study comprised 3011 patients.

The patients received either 400 mg of bezafibrate retard or placebo once a day. Patients continued their prescribed medications for cardiac and other conditions except lipid lowering drugs. The primary endpoint of the BIP study was fatal or non-fatal MI or sudden death (combined major cardiovascular events).

Cancer incidence data were obtained by matching a subject's personal identification number (PID) with the Israel National Cancer Registry (INCR). Each matched record was checked for correct identification.

The detailed description of INCR has been published previously [16]. In summary, the INCR is a population-based central tumor registry established in 1960 and since 1982 reporting to the registry is mandatory [17]. All medical facilities, both public and private and pathology laboratories that are diagnosing or treating cancer patients send a copy of their medical summary which contains tumor characteristics to the Registry. The INCR also collects data on cancer deaths from District Health Authorities and the Central Population Registry. In Israel, all demographic data are stored in the Central Population Registry in accordance with PID. The INCR is linked to this Registry and each cancer patient's personal data are then retrieved and validated. The last audit of data completeness concluded that registration was above 95% [18].

The mean follow-up period of the BIP study was 6.2 ± 0.8, range 4.7 to 7.6 years. The trial was conducted independently of the sponsor (Boehringer Mannheim GmbH, which is now part of F. Hoffmann-La Roche, Ltd), and it was approved by the Helsinki Committees of each center and the central national Helsinki Committee.

Data were analyzed using the SAS software, version 8.2 (SAS Institute Inc., Cary, NC, USA). Continuous variables at baseline were presented as mean values ± standard deviation (SD). Comparisons between groups were made using chi-square tests for discrete variables and Student t-test or Wilcoxon rank sum test for continuous variables. A *p*-value of less than 0.05 was considered as statistically significant.

Kaplan-Meier curves were produced using the LIFETEST procedure. The log-rank test was used for comparing the curves.

Multivariable analysis of the incidence of colon cancer was performed using the Cox proportional hazard model (PHREG procedure) to account for differing lengths of follow-up and correlation with covariates. Hazard ratio (HR) and 95% confidence interval (CI) for new colon cancer was calculated. Variables included in the models were age, gender, total cholesterol, ln transformed triglycerides, smoking status, study medication and body mass index (BMI).

## Results

### Baseline data

Our population included 2 groups: 1) Bezafibrate group – 1506 patients; 2) Placebo group – 1505 patients.

Patients in the placebo and bezafibrate groups were well balanced in terms of clinical and laboratory baseline characteristics (Table 1). The study groups were similar in regard to age, gender and the prevalence of the most relevant cardiovascular diseases and risk factors (a myocardial infarction in the past, hypertension, diabetes, heart failure, anginal syndrome). The majority of the patients in all groups were men who had sustained a myocardial infarction in the past. No significant differences between the groups were found for all types of cholesterol, systolic and diastolic blood pressure, heart rate, body mass index, fasting glucose, triglycerides and fibrinogen levels. At baseline, nitrates, calcium antagonists, beta blockers and antiplatelet drugs (mainly aspirin) were the most commonly used medications. There were no significant differences between the groups in the proportion of patients receiving other cardiovascular drugs.

**Table 1:** Baseline characteristics of the study population.

***Characteristics***	**Bezafibrate **(n = 1506)	**Placebo **(n = 1505)	**p value**
Age (years)	60.0 ± 6.8	60.0 ± 6.7	0.9
Body mass index (kg/m2)	26.7 ± 3.3	26.7 ± 3.3	0.7
Men (%)	1374(91)	1382 (92)	0.6
Past myocardial infarction (%)	1184 (79)	1163 (77)	0.3
Angina (%)	848 (56)	876 (58)	0.3
NYHA Class ≥2 (%)	45 (27)	47 (26)	0.9
Hypertension (%)	462 (31)	507 (34)	0.1
Current smokers	177 (12)	184 (12)	0.7
Systolic blood pressure (mmHg)	134 ± 18	133 ± 18	0.3
Diastolic blood pressure (mmHg)	81.1 ± 9.0	80.8 ± 9.1	0.4
Heart rate (beats/min)	70.1 ± 9.3	70.0 ± 9.3	0.8
Total cholesterol (mg/dl)	211 ± 17	213 ± 18	0.2
HDL-cholesterol (mg/dl)	34.5 ± 5.5	34.6 ± 5.5	0.7
LDL-cholesterol (mg/dl)	148 ± 16	149 ± 16	0.2
Triglycerides (mg/dl)	145 ± 51	145 ± 51	0.9
Fibrinogen (mg/dl)	350 ± 72	351 ± 74	0.7

### Clinical outcomes

**Figure 1:**
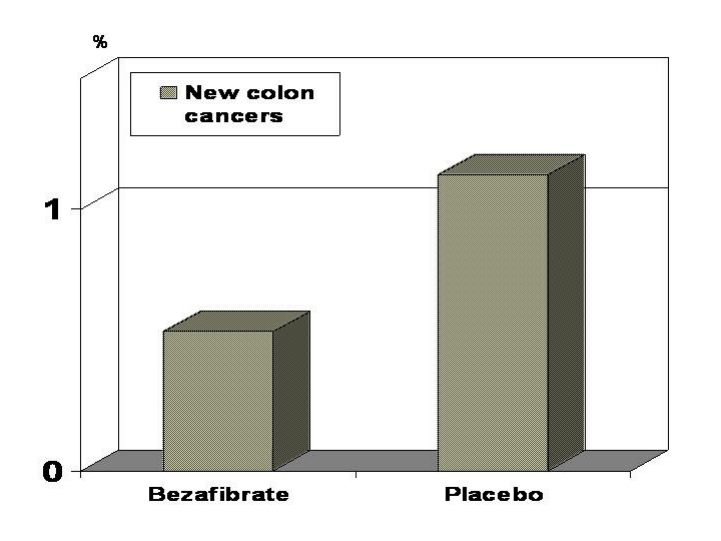
New colon cancers (%) during follow-up; bezafibrate vs. placebo (Fisher's exact test: one side p = 0.05; two side p = 0.07).

During the follow-up period, development of new cancer (all types) was recorded in 177 patients: in 79 (5.25%) patients from the bezafibrate group vs. 98 (6.51%) from the placebo group (p = 0.14). Development of new colon cancer was recorded in 25 patients (Figure 1): in 8 (0.53%) patients from the bezafibrate group vs. 17 (1.13%) from the placebo group, (Fisher's exact test: one side p = 0.05; two side p = 0.07).

**Figure 2:**
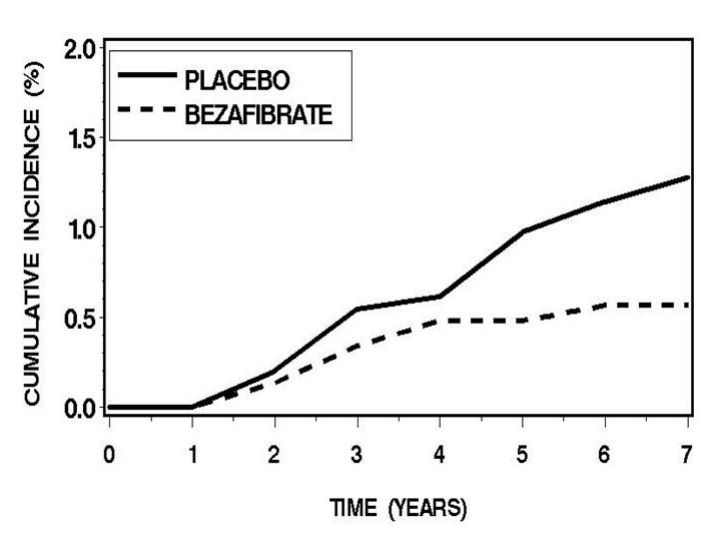
Kaplan-Meier curves of colon cancer incidence (in accordance with the time of diagnosis) for the study groups (bezafibrate vs. placebo; 6.2 years mean follow-up, p log-rank = 0.07).

Kaplan-Meier curves of colon cancer incidence (in accordance with the time of diagnosis) for the two study groups are presented in Figure 2. The incidence rate of patients on placebo tended to be higher than in their bezafibrate treated counterparts, (p log-rank = 0.07). A difference in the incidence of cancer was only detectable after a 4 year lag and progressively increased with continued follow-up.

On multivariable analysis the colon cancer risk in patients received bezafibrate tended to be lower with a hazard ratio of 0.47 and 95% confidence interval 0.2–1.1.

## Discussion

The main novel finding in this *post hoc *analysis of the BIP study, which was randomized and double blind in design, is a possible preventive effect of bezafibrate on the development of colon cancer in humans. The study hypothesis is supported not only by the magnitude of colon cancer risk reduction in patients treated by bezafibrate (53%), but, more importantly by the time-course of the Kaplan-Meier curves: The 4 year lag in the occurrence of detectable differences between groups, followed by a progressive disparity in colon cancer incidence between patients on bezafibrate and patients on placebo are characteristic of a biologic effect and thus support causality rather than chance association.

Accumulating animal experimental, human laboratory and epidemiologic data [6,10-14,19,20] support the hypothesis linking triglyceride levels and insulin resistance to the development of colon cancer. These facts emphasize the potential for this cancer to become a preventable disease not only via screening and removal of polyps but through relevant lifestyle changes and pharmacological interventions which can provide even more avenues for prevention [19-25].

The fact that the incidence of insulin resistance has been increasing in the Western world where colon cancer is the second leading cause of cancer death makes the exploration of the interrelationship of these conditions a subject of high priority for public health.

The biological role of the peroxisome proliferator-activated receptors (PPARs) in various diseases, including inflammation and cancer, has been highlighted recently [8-14,26-28]. PPARs are members of the nuclear hormone receptor family of ligand-activated transcription factors that play a prominent role in the regulation of many metabolic processes. The alpha and gamma isoforms of PPAR are important regulators in lipid and glucose metabolism, cell differentiation and inflammatory response [29,30]. These data propose that PPAR alpha, beta/delta and gamma may be associated with many aspects of colon cancer development including insulin- and inflammation-related mechanisms.

The fibric acid derivative bezafibrate is the pan – (alpha, beta/delta, gamma) PPAR activator with predominantly PPAR alpha (as all fibrates) and beta/delta effects but also with perceptible PPAR gamma properties [31-36]. The use of bezafibrate is associated with triglyceride-lowering and HDL-cholesterol raising effects resulting in decreased systemic availability of fatty acid, diminished of fatty acid uptake by muscle and improvement of insulin sensitization [37,38]. These direct and indirect effects may have contributed to the suppression of the development of colonic tumors in rodents by bezafibrate [12-14].

### Limitations of the study

Although development of new colon cancer in patients randomized to bezafibrate was more than half as frequent as in those randomized to placebo, the difference reached only borderline statistical significance. The BIP study was designed (both in terms of sample size and time of follow-up) to detect the effect of the lipid-modifying agent bezafibrate on major cardiovascular events in coronary patients at high risk, but had not an appropriate statistical power to explore development of such a relatively low-incidence disease as colon cancer. In addition, caution should be used in interpreting our findings, as they were identified in post-hoc analysis.

However, the detected pattern of the time course in the differences between groups, suggesting a biologically meaningful effect, is encouraging and supports the initiation of a large prospective controlled trial in light of its importance for public health.

## Conclusion

The use of bezafibrate as lipid-modifying agent in patients with coronary artery disease appears to be associated with a reduced risk of colon cancer.

## Competing interests

The authors declare that they have no competing interests.

## Authors' contributions

AT conceived the study and drafted the manuscript, EZF, YA and SB were involved in the study design, coordination and data acquisition, AT, VB and SB studied and matched the records from the Cancer Registry, EZF, IG, ES, JS and MSF interpreted the results and VB performed the statistical analysis of the data presented, EZF, DT, SB, ES, MSF and MM critically reviewed the study for important intellectual content. All authors approved the final version of the manuscript.
